# Activity of Aqueous Extracts from Native Plants of the Yucatan Peninsula against Fungal Pathogens of Tomato In Vitro and from *Croton chichenensis* against *Corynespora cassiicola* on Tomato

**DOI:** 10.3390/plants11212821

**Published:** 2022-10-24

**Authors:** Felicia Amalia Moo-Koh, Jairo Cristóbal-Alejo, José María Tun-Suárez, Irma Leticia Medina-Baizabal, Alejandra Anahi Arjona-Cruz, Marcela Gamboa-Angulo

**Affiliations:** 1 Centro de Investigación Científica de Yucatán, Calle 43 No. 130, Col. Chuburná de Hidalgo, Yucatán, Mérida 97205, Mexico; 2 Tecnológico Nacional de México, Campus Conkal, Avenida Tecnológico s/n, Yucatán, Conkal 97345, Mexico

**Keywords:** Alternaria alternata, biocontrol, Bonellia flammea, Curvularia lunata, Fusarium equiseti, leaf spot severity, plant extracts

## Abstract

Plant extracts are a valuable alternative to control pathogens of horticultural crops. In the present study, four species of pathogenic fungi were isolated from leaf spots on *Solanum lycopersicum* and identified by traditional and molecular techniques as *Alternaria alternata* ITC24*, Corynespora cassiicola* ITC23, *Curvularia lunata* ITC22, and *Fusarium equiseti* ITC32. When 11 aqueous extracts from eight native plants of the Yucatan Peninsula were tested against the four fungi in vitro, the extract from *Croton chichenensis* roots was most active, inhibiting mycelial growth (79–100%), sporulation (100%), and conidial germination (71–100%) at 3% (*w*/*v*). A logarithmic–diagrammatic scale of the pathosystem *C. cassiicola–S. lycopersicum* was established and used to assess disease severity on inoculated tomato plants in a greenhouse after treatment with the aqueous extract from *C. chichenensis* roots at 12% (*w*/*v*). After 21 days, the disease severity was 57% lower than on the control without extract applied. This dose of the extract was not phytotoxic to tomato leaves and was compatible with the beneficial organisms *Bacillus subtilis* CBCK47 and *Trichodema asperellum* Ta13-17. The antifungal efficacy of *C. chichenensis* is highly promising for incorporation into integrated disease management of tomato crops.

## 1. Introduction

Phytopathogens are an ongoing threat to plant production because they often persist and evade plant defenses, causing disease and global annual losses of billions of US dollars. To control fungal plant diseases, farmers have used synthetic fungicides that suppress the fungi; however, misuse of these products results in the development of resistance and adverse environmental effects [1,2] on beneficial soil organisms and pollinating insects, other organisms, and humans. The use of natural plant products is thus receiving increased interest from farmers who are focusing on more sustainable production and helping reduce the risk of resistance development in a pathogen [3,4,5]. 

Plants produce secondary metabolites such as alkaloids, flavonoids, terpenes, phenols, glycosides, tannins, and fatty acids that inhibit the growth of bacteria and fungi. These metabolites can be located in two groups, phytoanticipins and phytoalexins. Phytoanticipins are constitutively present in the plant, and phytoalexins are biosynthesized in response to pathogen attacks [5]. Thus, plant extracts frequently contain a mixture of compounds with different modes and mechanisms of action. In addition, some compounds may act synergistically to inhibit the growth of phytopathogens [4,6]. This effect of a mixture of compounds is also known as compatibility between natural products produced by the same or different organism species [1,5]. However, plant extracts can also affect seed germination and plant growth and be toxic to beneficial organisms. For example, an aqueous extract of *Seriphidium kurramense* had a toxic effect on *Lemna minor* at different concentrations (10, 100, and 1000 µg/mL) [7]. An extract of *Cleome arabica* L. had a toxic effect on seedlings of *Lactuca sativa*, *Raphanus sativus*, *Peganum harmala*, and *Silybum marianum* [8]. Therefore, the toxicity of the extracts must be evaluated before they can be applied to a crop.

With 2330 native vascular plant species, the Yucatán Peninsula represents 10% of Mexico’s biodiversity [9,10]. In previous studies and following chemotaxonomic, medicinal, or serendipity criteria, plant species from this tropical region were collected and tested against plant pathogenic fungi [11,12,13,14,15]. *In vitro* tests of extracts from *Acacia pennatula*, *Acalypha gaumeri*, *Bonellia flammea, Calea jamaicensis, Croton chichenensis*, *Licaria* sp., *Mosannona depressa, Parathesis cubana,* and *Piper neesianum* showed antifungal activity [11,12,13,14,15]. Among these extracts, however, only those from *A. gaumeri* and *B. flammea* were effective in vivo against *Alternaria chrysanthemi*, the causal agent of early blight on *Chrysanthemum morifolium* [16]. 

*Corynespora cassiicola* is a phytopathogenic fungus that induces irregular spots on the leaves of more than 530 plants, including tomato (*Solanum lycopersicum*) [17] among 380 genera in the tropics and subtropics, including numerous economically important crops [18]. The disease incidence in fields can reach 60%. Other phytopathogenic fungi on tomatoes are *Alternaria solani*, *A. tenuis*, *Cladosporium fulvum*, *Colletotrichum phomoides*, *Phytophthora infestans*, *P. nicotianae* var. *parasitica*, *Phoma destructiva*, *Curvularia* spp., and *Fusarium* spp. [19,20,21,22]. Identifying the presence of *C. cassiicola* is difficult due to the existence of fungal complexes and secondary parasites that take advantage of the lesions to establish themselves [23]. Tomato leaf spots can affect from 35–58% up to 80% of the plants in greenhouses, so tools to monitor and identify this phytopathogen are essential [24,25]. One such tool that has been developed to monitor phytopathogens in the field and greenhouse is the logarithmic–diagrammatic scale that allows the monitoring of the early symptoms and estimates the intensity of the disease of *C. cassiicola* in tomato [26,27].

In the present study, we isolated four fungi from tomato leaf spots and confirmed that they caused the leaf spots. We then selected eight native plants previously reported with antifungal effects on plant pathogens, and their aqueous extract assessed in vitro activity against the isolated fungi. These aqueous extracts (11) were from different parts of *A. gaumeri*, *B. flammea*, *C. chichenensis*, *C. jamaicensis*, *Licaria* sp., *M. depressa*, *P. cubana*, and *P. neesianum*. We then developed a logarithmic–diagrammatic scale for the early detection of the symptoms induced by *C. cassiicola*, tested the most effective aqueous extract against the fungus on tomato plants, and evaluated its phytotoxicity and compatibility with beneficial organisms.

## 2. Results

### 2.1. Identification of Fungal Phytopathogens 

**Figure 1:**
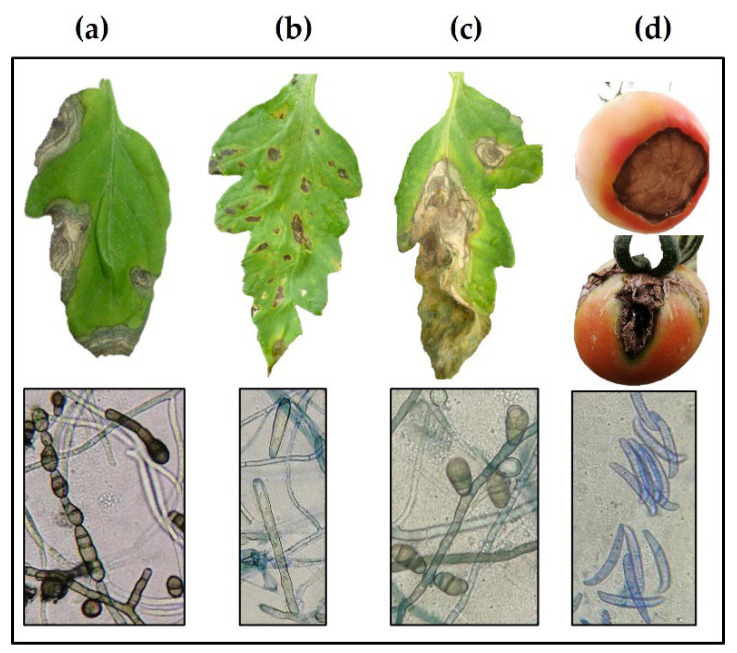
Fungal phytopathogens and symptoms induced on *Solanum lycopersicum*. (**a**) *Alternaria alternata* ITC24, (**b**) *Corynespora cassiicola* ITC23, (**c**) *Curvularia lunata* ITC22, and (**d**) *Fusarium equiseti* ITC32.

Three fungal strains belonging to the genera *Alternaria* (strain ITC24), *Corynespora* (strain ITC23), and *Curvularia* (strain ITC22) were isolated from the adult leaves with dry, circular to oval, chlorotic, and necrotic spots, the typical symptoms of leaf spots on tomato. In addition, *Fusarium* (strain ITC32) was isolated from tomato fruit with a brown rot at the base, which expanded over about 50% of the fruit surface. According to Koch’s postulates, pathogenicity tests verified that the isolated fungi were responsible for the disease in the *S. lycopersicum* cultivars (Figure 1). 

The four phytopathogenic fungal isolates were morphologically and molecularly identified as *Alternaria alternata* (Fr.) Keissl strain ITC24, *C. cassiicola* Berk. & M.A. Curtis strain ITC23, *Curvularia lunata* (Wakker) strain ITC22 and *F. equiseti* (Corda) Sacc. strain ITC32 (Table 1). The DNA extracted from each fungal strain was amplified by PCR using universal primers ITS1 and ITS4. The sequencing of the amplified PCR products showed between 400 and 600 bp in length. Comparison of the edited sequences on the Bioedit software for each fungal strain with NCBI sequences showed 99–100% similarity, which were deposited in GenBank (Table 2).

### 2.2. Antifungal In Vitro Activity of Aqueous Extracts 

**Figure 2:**
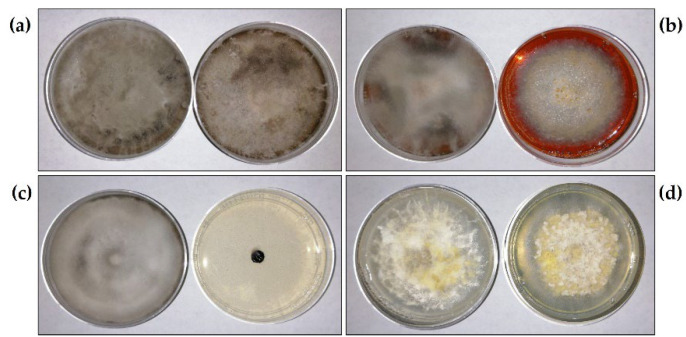
Effect of aqueous extract from *Croton chichenensis* roots at 3% (*w*/*v*) on mycelial growth of (**a**) *Alternaria alternata* ITC24, (**b**) *Corynespora cassiicola* ITC23, (**c**) *Curvularia lunata* ITC22 and (**d**) *Fusarium equiseti* ITC32 in dilution agar assay, left control, right extract.

After 7 days at 3% (*w*/*v*) concentration, the evaluated aqueous extracts significantly inhibited at least one of the fungal variables assessed for the four pathogens in relation to the control (*p* ≤ 0.001). The most active aqueous extract was from *C. chichenensis* root and caused 99–100% inhibition of mycelial growth (IMG) of *C. cassiicola* ITC23, *C. lunata* ITC22, and *F. equiseti* ITC32. The next most active was the aqueous extract from the stem bark of *B. flammea*, with 93.7% IMG of *C. lunata* (Table 2, Figure 2). The aqueous extracts of *M. depressa* stem bark were less active (19.5–48.9% IMG) against the four pathogens. The remaining eight extracts had no significant (0–28.7% IMG) effect against the pathogens assayed (Table 3). 

The total inhibition of sporulation (IS, 100%) of all four pathogenic fungi was provided by the aqueous extract from *C. chichenensis* roots, from *B. flammea* bark stem *A. alternata* ITC24 and *C. lunata* ITC22 (83.4 and 93.3% against the other two), from *A. gaumeri* roots and *Licaria* sp. stem bark against *A. alternata* ITC24 (100%), *C. lunata* ITC22 (100, 96.8%, respectively), and *F. equiseti* ITC32 (89.9 and 100%, respectively) at 3% (*w*/*v*) after 7 days. Except for the aqueous extract from *P. cubana*, the six remaining extracts inhibited the sporulation of two phytopathogenic fungi to varying extents (IS = 10.5–100%) (Table 4). 

**Figure 3:**
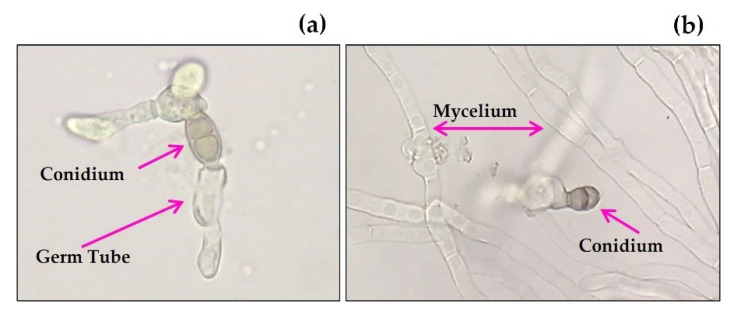
Effect of (**a**) aqueous extract from roots of *Croton chichenensis* at 3% (*w*/*v*) after 5 h on conidial germination of *Curvularia lunata* ITC22, (**b**) control (water).

For the inhibition of conidial germination (ICG), the most active aqueous extract was from *C. chichenensis* roots at 3% (*w*/*v*), and the germination was inhibited in a range of 70.5 to 100% for the four phytopathogenic fungi after 5 h of exposure. The aqueous extract from *M. depressa* root bark caused less but significant ICG against *F. equiseti* ITC32 (61.3%), *C. cassiicola* ITC23 (80.1%), and *C. lunata* ITC22 (100%). The conidia of *C. cassiicola* ITC23 were most sensitive to the aqueous extracts evaluated, and germ tubes on conidia that germinated were often deformed (Table 5, Figure 3).

The aqueous extract of the roots from *C. chichenensis* had the greatest activity against the four phytopathogenic fungi in the sporulation and germination and completely inhibited the mycelial growth of *C. cassiicola* ITC23, *C. lunata* ITC22, and *F. equiseti* ITC32.

### 2.3. Corynespora cassiicola–Solanum lycopersicum Pathosystem

#### 2.3.1. Logarithmic–Diagrammatic Scale Development

**Figure 4:**
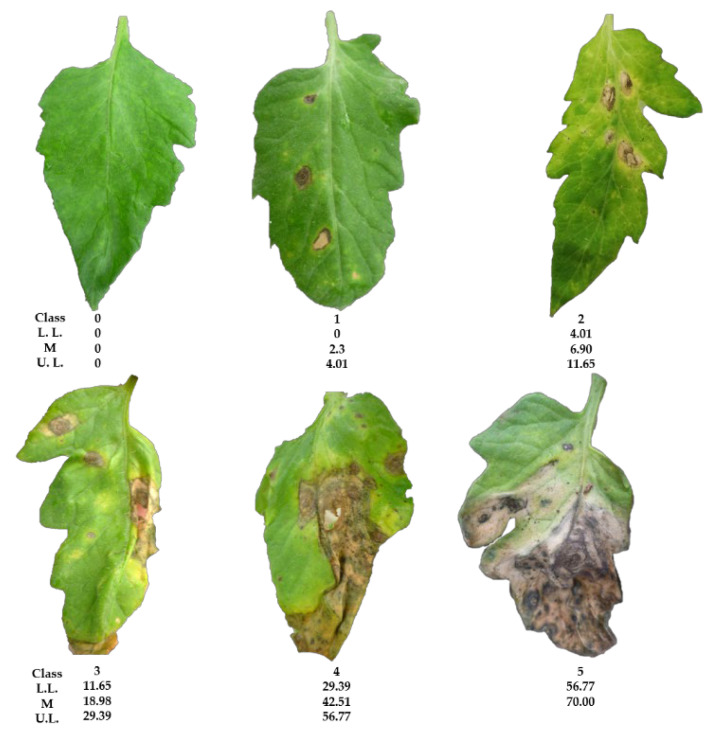
Logarithmic–diagrammatic scale of severity of the leaf spot disease in the pathosystem *Solanum lycopersicum–Corynespora cassiicola* using the software 2LOG. Classes: 0–5, L.L. = Lower Limit (%), M = Midpoint (%) and U.L. = Upper Limit (%).

The diseased leaves (*N* = 42) from tomato plants were photographed to calculate the leaf-spot area on each leaf and develop a logarithmic–diagrammatic scale to assess the disease severity. The largest calculated area (56.1 pixels) was equivalent to an estimated 70.0% of disease severity or dead surface. Six classes (0–5) were established, with class zero corresponding to leaves without symptoms (apparently healthy) and class 5 to leaves with severe (midpoint = 70.0%) major damage. Classes 1–4 values correspond to the midpoints with lower disease severity of 2.3, 6.9, 18.0, and 42.5%, respectively, were obtained using the software 2LOG. Finally, a representative image from each class was selected as part of constructing the scale of the pathosystem *C. cassiicola–S. lycopersicum* (Figure 4).

#### 2.3.2. Validation of Logarithmic–Diagrammatic Scale 

The first validation of the logarithmic–diagrammatic scale based on the six severity classes for the pathosystem *C. cassiicola*–*S. lycopersicum* showed a precision of 0.79 (*r*^2^) and an accuracy of 0.81 (*b*_1_); for the second validation, these values were 0.83 and 0.85, respectively. With these results, the scale was used to estimate leaf-spot severity and the efficacy of disease control using the plant extracts. 

### 2.4. In Vivo Effect of Extract from Croton chichenensis on Tomato Leaf Spot

The severity scale for leaf spots on the tomato plants showed that the plants treated with the aqueous extract of the *C. chichenensis* root significantly (*p* ≤ 0.001) reduced leaf spot area compared with the untreated controls. The area under the disease progress curve (AUDPC) showed a lower percentage of lesion area (64.56% for day) in the treatment with the 12% (*w*/*v*) aqueous extract from *C. chichenensis* roots and the lowest final severity (*Y*_final_ = 10.43%). The progress of the apparent infection rate obtained from the reciprocal of b parameter of the Weibull model with the aqueous extract showed that leaf spot severity was very low (0.01901%/day) compared with the untreated control, which had the highest intensity (0.03012%/day) of the disease (Table 6).

**Figure 5:**
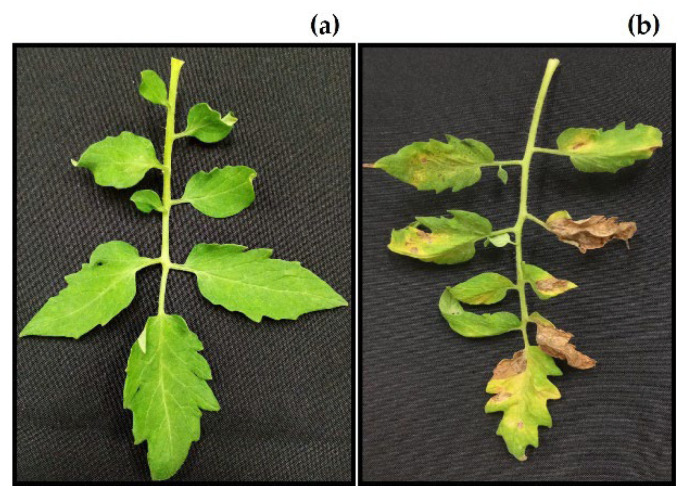
Efficacy of the (**a**) aqueous extract of *Croton chichenensis* (12%, *w*/*v*) and (**b**) control (water) in the biocontrol of leaf spot on *Solanum lycopersicum* 21 days after inoculation with *Corynespora cassiicola* ITC23.

The leaves of the control plants had significant concentric spots and chlorosis by 7 days after inoculation with *C. cassiicola*, whereas the leaves on the plants treated with the aqueous extract from *C. chichenensis* roots were green and had no symptoms. At 21 days after inoculation, most of the leaves on the control plants had great (Class 5) disease severity. In contrast, the treated plants still had no symptoms (Class 0) (Figure 5).

### 2.5. Phytotoxicity of Croton chichenensis Roots

**Figure 6:**
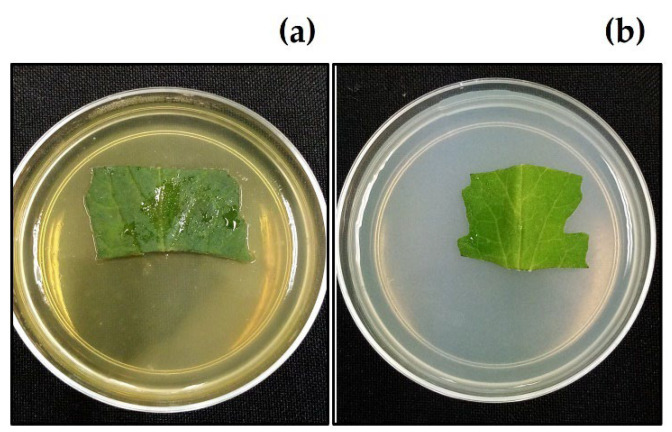
*Solanum lycopersicum* fragment leaves from phytotoxicity test after 7 days on agar with exposition to (**a**) aqueous extract from *Croton chichenensis* roots at 12% (*w*/*v*) and (**b**) control (water).

After 7 days on agar containing 12% (*w*/*v*) aqueous extract from *C. chichenensis* roots, the leaves of *S. lycopersicum* had no necrosis or visible damage to the upper surface, veins, and leaf margin and no hypersensitivity (Figure 6).

### 2.6. Compatibility of Croton chichenensis Extract and Beneficial Organisms

**Figure 7:**
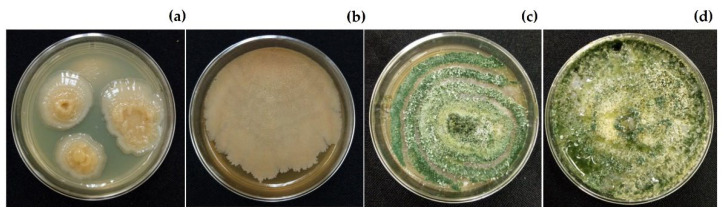
The aqueous extract from *Croton chichenensis* roots (12%, *w*/*v*) had compatibility with (**a**) *Bacillus subtillis* CBCK4, (**b**) control (bacteriological agar medium), (**c**) *Trichodema asperellum* Ta13-17, and (**d**) control (potato dextrose agar medium).

The aqueous extract of *C. chichenensis* roots 12% (*w*/*v*) had no statistically significant differences (*p* ≤ 0.001) with the control on the compatibility assay with beneficial organisms. After 2 days of exposure to the aqueous extract, three colonies of *Bacillus subtilis* CBCK47 were detected on bacteriological agar medium and grew to a diameter of 2–3 cm in 5 days. The plant growth-promoting organisms *Trichoderma asperellum* Ta13-17 grew 8 cm in diameter, and conidial was detected on potato dextrose agar (PDA) after 7 days of exposure to the aqueous extract (Figure 7). This extract of *C. chichenensis* in the medium slightly modified the growth but did not inhibit both organisms, then had 100% compatibility.

## 3. Discussion

Here, we identified four fungi causing leaf spot diseases in tomato and tested the efficacy of aqueous extracts from native species of the tropical Yucatan Peninsula against these fungi as part of our goal to conserve and take advantage of the regional biodiversity. The morphological identification of the four phytopathogens showed similarity with the morph length and width of the conidial previously reported in the literature [28,29,30,31,32,33,34] (Table 1), which is supported by molecular identification with an admisible percentage of similarity [34]. Genetic studies of species have become increasingly valuable as they complement each other by helping to overcome the difficulties of using traditional morphological identification alone [23,35,36,37]. 

Tomato leaf spot has been reported in other countries since late 1986 [38,39]. Tomato leaf spot has been attributed to the presence of the fungi *Cladosporium cladosporioides* and *C. fulvum* [20,40], *Stemphylium lycopersici* [41], and *C. cassiicola* [24]. Among these pathogens, *C. cassiicola* has the most hosts [18]. In Mexico, *C. cassiicola* has been isolated from the leaves of *Capsicum annuum* L., *C. chinense* Jacq. [13,42], *Hibiscus sabdariffa* L. [43,44], *Gossypium hirsutum* L. [45] and *Vicia faba* L. [46]. In the present study, *C. cassiicola* ITC23 (Corynesporascaceae) was found together with *A. alternata* ITC24 and *C. lunata* (Pleosporaeae)*,* affecting tomato at the foliar level [28]. These three microorganisms form fungal complexes, making their identification difficult [19,47,48]. All three have dark-coloured colonies and conidia and free-form conidiophores [13,49]. *A. alternata* is known to cause tomato leaf blight [50], and *C. lunata* causes leaf spots on pepper (*Capsicum annuum*) [51], but we found no reports of *C. lunata* infecting tomato. 

In the present study, *F. equiseti* ITC32 formed sporodochia as asexual fruiting structures containing conidia on free conidiophores on tomato fruits [52]. The fungus is cosmopolitan; it has a wide host range and easily adapts to different climatic environments [53,54]. It is also associated with tomato wilt; infection begins in the roots and impacts all plant parts [55]. However, wilt and rot of tomato fruits are associated with *F. oxysporum* [22,56], but we found no reports of *F. equiseti* causing a disease of tomato fruits. Fulfilling Koch's postulates confirmed that the mycelium and spores of the fungus and symptoms were caused by *F. equiseti* in tomato fruits after infection.

Aqueous extracts from plants with bioactive properties against plant pathogens have been described and used worldwide, and more than 2500 species from 235 plant families have been identified as effective against pests [57,58]. As expected, 91% of the aqueous extracts evaluated, except that from *P. cubana*, had an inhibitory effect greater than 50% against the four pathogens for at least one of the variables measured. The most active aqueous extract that inhibited mycelial growth, sporulation, and conidial germination (70.5% to 100%) was from the roots of *C. chichenensis* against *C. cassiicola* ITC23, *C. lunata* ITC22, and *F. equiseti* ITC32; and completely inhibited the sporulation of *A. alternata* ITC24 (100%). On the other hand, the aqueous extract of *B. flammea* stem bark greatly inhibited sporulation in the four organisms, but it only affected the inhibition of conidial germination of *C. lunata* ITC22 and *F. equiseti* ITC32. In a previous report, an aqueous extract from *B. flammea* (3%, *w*/*v*) showed the inhibition of mycelial growth of 50%, conidial germination of 98%, and no effect on the sporulation of *C. cassiicola* ITC7, fungus isolated from *Citrus chinense*; and a good effect on the three variables (58–89%) on *C. lunata* ITC4, fungus isolated from *Thrinax radiata* [13]. Total or partial inhibition of the mycelial growth of pathogenic fungi with plant extracts is important, but sporulation and germination inhibition tests are variables considered fundamental before further developing a natural fungicide. Aqueous extracts of plants can control common fungi that infest plants and produce conidia [59]; inhibiting conidial germination, which would prevent new infections, is highly desirable for an aqueous extract [60,61]. In our study, the aqueous extract from *C. chichenensis* root provided the highest inhibition of conidial germination. 

The species *C. chichenensis* is an endemic shrub to the Yucatan Peninsula and belongs to the Euphorbiaceae family with a great diversity of species [9]. Species in the genus produce numerous compounds with potential pharmacological use, including 339 diterpenes, seven sesquiterpenes, one sesterterpene, one triterpene, 21 glycosides, eight alkaloids, three benzoate derivatives, three pyran-2-one derivatives, two cyclopeptides, two propane derivatives, and two limonoids, among others. The activity of ethanolic extracts compared to the aqueous extracts is related to the presence of more polar metabolites such as saponins and other glycosides alkaloids, flavonoids, and tannins, among others [62].

In previous studies, the ethanolic extract from *C. chichenensis* roots inhibited the mycelial growth of *A. tagetica* ATTC53771, *C. gloeosporioides* CICY002, *F. oxysporum* CICY003, and *Rhizopus* sp. CICY004 at 2 mg/mL in a dilution agar assay [10]. Compounds from other *Croton* species also have antifungal activity. For example., the ethanolic extract from *C. leptostachyus* stem and roots inhibited the growth of *F. oxysporum* (IC_50_ of 9726 and 1133 mg/L, respectively) in a dilution agar assay [63]; an ursane-type triterpenoide from *C. bonplandianum* roots inhibited *A. alternata*, *Colletotrichum camellie*, *C. gloeosporioides*, *Curvularia eragrostidies*, and *F. equiseti* (IC_50_ of 10–15 µg/mL) [64]; and an ethanolic extract from *C. heliotropiifolius* stem bark inhibited *Candida albicans* at 2.5 mg/mL in a bioautographic assay [65]. Thus, our report is the first antifungal effects of aqueous extracts from *C. chichenensis* roots against *A. alternata*, *C. cassiicola*, *C. lunata*, and *F. equiseti*.

To evaluate the antifungal effect of the aqueous extract from *C. chichenensis* on plants in the greenhouse, we first developed a logarithmic–diagrammatic scale for the pathosystem *C. cassiicola*-*S. lycopersicum* to estimate disease severity. The precise values that we obtained and those of the reported scales demonstrate that the scales are good tools for estimating the severity of the pathogens. For example, the diagrammatic scale for *C. annuum*–*Cercospora* sp. had a range of 0.79 to 0.95 [66],0.61 to 0.95 for *Annona cherimola*–*C. gloeosporioides* [26], and 0.81 to 0.92 for the Tar Stain Complex in Maize [27]. 

In controlling leaf spots in tomato, the aqueous extract of *C. chichenensis* roots reduces the speed of symptom development over time, resulting in a large difference in severity. In other greenhouse studies, an aqueous extract of *Ocimum basilicum* at 10% (*w*/*v*) reduced by 43% the calculated rate of disease caused by *C. cassiicola* [67]; an aqueous extract of cinnamon at 0.25% (*w*/*v*) reduced the severity of grapevine downy mildew caused by *Plasmopara viticola* by 67% [68]. Volatile metabolites from the leaves of *Solanum habrochaites* (LA1777) reduced the severity of the late blight of tomato caused by *Phytophthora infestans* EG_7 by 97% and lowered the AUDPC [69]. In a field test using the pathosystem *Chrysanthemum morifolium*–*A. chrysanthemi*, an aqueous extract from *A. gaumeri* roots and *B. flammea* stem bark at 3% (*w*/*v*), reduced disease severity by 67% and 50%, respectively, and lowered AUDPC [16].

The toxicity tests of promising candidates are mandatory to evaluate their effects on aerial parts of plants, seeds, beneficial organisms, and human cells. These studies also provide valuable information for future evaluations of the application of plant extracts in the field [7,70,71]. In this study, *B. subtilis* and *T. asperellum* were selected for their history of promoting plant growth, other benefits, and their biological plasticity [72,73]. No symptoms of toxicity were apparent on tomato leaves or the beneficial organisms evaluated after treatment with the 12% (*w*/*v*) aqueous extract of *C. chichenensis* roots, which is compatible for its possible combination and application with the biocontrol agents *Trichoderma* and *Bacillus* [74,75]. By contrast, *in vitro* assays showed that the aqueous extracts from *Citrus chinense* fruits at 50% (*w*/*v*) [76] inhibited the population growth of *E. coli* and *Bacillus* sp., and the aqueous extracts from *Phyllantus* sp., *Azadirachta indica*, and *Tagetes patula* inhibited the growth of *Trichoderma* and *Metarhizium* at 15% (*w*/*v*) [77]. 

The activity of the aqueous extract of *C. chichenensis* at 12% is promising against tomato pathogens, but its activity needs to be tested on other tomato accessions and pathogens and in the field in different conditions. This promising extract has the potential to be combined with beneficial organisms such as growth promoters and biocontrol agents in an integrated management program of tomato diseases. The active compounds in *C. chichenensis* roots also need to be isolated and identified.

## 4. Materials and Methods

### 4.1. Isolation and Pathogenicity Tests of Phytopathogenic Fungi

The four phytopathogenic fungi used in the present study were isolated from *S. lycopersicum* plants grown at the Tecnológico Nacional de México/Campus Conkal (Table 1 and Table 2). The leaf and fruit samples with apparent fungal-induced lesions were cut into small fragments (0.2–0.5 cm^2^) and surface-sterilized with NaClO at 2%, then washed twice with sterile distilled water and blotted dry with sterile absorbent paper. The pieces were placed on potato dextrose agar (PDA) in Petri dishes and kept at 28 ± 2 °C with 12 h light/12 h dark for 7 days until fungal colonies were observed [13]. The colonies had different colours and were purified by transferring the mycelium to PDA. 

The pathogenicity of the purified fungi as the cause of the plant symptoms was confirmed using Koch's postulates by spray inoculating healthy leaves and fruits of tomato with a conidial suspension (1 × 10^6^ conidia/mL) of fungi [49]. Each fungal species produced the original symptoms on the host and was re-isolated and purified as described above. 

### 4.2. Identification of the Fungi

The four purified pathogenic fungi were morphologically identified to the genus level using the dichotomous keys of Barnett and Hunter [78] and Manamgoda et al. [79]. The DNA was extracted from the mycelium of the pure fungal strains grown on PDA after three days and using the methods of Moo-Koh et al. [13] and the ZR Fungal/Bacterial DNA MiniPrep Kit (Zymo Research, Irvine, CA, USA). ITS1 and ITS2 between ribosomal genes (rDNA) 18S–5.8S and 28S were amplified by PCR using primer pair ITS1 (5′ CCGTAGGTGAACCTGCGG 3′) and ITS4 (5′ TCCTCCGCTTATTGATATGC 3′) (Integraded DNA Technologies–IDT, Coralville, IA, USA) [80]. The amplified products were sequenced (Macrogen, Inc., Seoul, South Korean), edited with the Bioedit software (https://bioedit.software.informer.com/7.1/: accessed on 21 June 2022), and compared with the data available in the BLAST (https://www.ncbi.nlm.nih.gov/genbank/: accessed on 22 June 2022). The sequences for each fungal species were deposited at the GenBank in National Center for Biotechnological Information (NCBI) (Table 2). 

### 4.3. Plant Material

The plant material of the eight selected species for aqueous extraction was collected from the Yucatan Peninsula by the working group of the Biotechnology Unit of the Centro de Investigación Científica de Yucatán (Table 7) [13,14,15].

### 4.4. Preparation of Aqueous Extracts

Dried, ground plant material (30 g/L) was transferred to a flask with 500 mL of boiling distilled water and stirred for 20 min. The mixture was cooled, then filtered through Whatman No. 1 filter paper, then through a 22 µm membrane (Millipore Merck, Burlington, MA, USA), and had a final concentration of 6% (*w*/*v*). Sterile filtrates were kept for 24 h at 4 °C until used in bioassays [13,15].

### 4.5. In Vitro Antifungal Bioassay of Aqueous Extracts

#### 4.5.1. Inhibition of Mycelial Growth

The filtered, sterilized aqueous extracts were evaluated in a PDA dilution test (BD, Bioxon, Estado de México, México). The PDA (3.9 g in 50 mL, 2:1 *w*/*v*) was sterilized and held at 50 °C for mixing with the aqueous extract to a final concentration of 3% (*w*/*v*), 10 mL was added to each plate and held at room temperature for 24 h. A mycelial disc (5 mm diameter) from a 6-to-7-day-old culture of the test fungus was placed in the middle of the dish. A Petri dish of medium without extract was used as a control. Five replicate plates were tested for each extract–fungus combination and incubated at 28 ± 2 °C with 12 h light /12 h dark [13]. The diameter of each colony was measured with a Vernier digital calliper every 24 h until the colony in control covered the agar surface. The efficacy of the extract at inhibiting mycelial growth was calculated using the Abbott formula [81], *E* (%) = (Control − Treatment)/Control)(100), where *E* = efficacy (%), Control = mycelial diameter of the control (cm), Treatment = mycelial diameter of the treatment (cm).

#### 4.5.2. Inhibition of Sporulation 

Sterile water (9 mL) was added to treatment and control plates in which the fungus grew horizontally and vertically, and the mycelial surface was gently scraped to remove the conidia. The suspension was filtered through two layers of sterile gauze, then a 1:9 (*v*/*v*) dilution of suspension to water was homogenized, and 9 µL was transferred to a storage Neubauer counting chamber. Conidia in four fields of view were counted with a light microscope (Leica model DM500, Wetzlar, Germany), and the total number of conidia/mL was calculated as NE = (*X*/0.1) (1000)(9), where *X* is the mean number of conidia in 4 fields, 0.1 is the depth of the chamber; 1000: volume (mL); 9: volume of water (mL) added to Petri dish. The values were used in the Abbott formula described above to calculate the inhibition of sporulation [81].

#### 4.5.3. Inhibition of Spore Germination

A 9 µL drop of the undiluted conidial suspension was dropped on each of the quadrants of a Petri dish containing PDA plus the aqueous extracts (3%, *w*/*v*). PDA alone was used as the control, and each quadrant was considered a replication. Conidia were considered germinated when a germ tube was observed. Conidia were checked for germination, and the germinated conidia were counted in each quadrant every 60 min for five h, to ensure the germination of the conidia in control, using a light microscope (Leica model DM500, Wetzlar, Germany) with a magnification of 400× (Figure 3).

#### 4.5.4. Statistical Analyses

The experimental design was completely randomized. The percentage data for the inhibition of mycelial growth, sporulation, and conidial germination by not complying with the assumptions of normality of these data were arcsine square-root-transformed before calculating an ANOVA. Tukey’s test (*p* ≤ 0.05) was used, and separation of means was carried out with the SAS ver. 9.4. for Windows (SAS Institute, Cary, NC, USA) for all analyses.

### 4.6. Pathosystem Corynespora cassiicola—Solanum lycopersicum 

#### 4.6.1. Development of the Logarithmic—Diagrammatic Scale 

The severity of *C. cassiicola* on the tomato crops was measured with a logarithmic–diagrammatic scale developed for this study using 42 photographs of tomato leaves with leaf spots caused by *C. cassiicola*. For each leaf, the total area with lesions was measured using the software ImageJ (National Institutes of Health, Bethesda, MD, USA) [82]. The total area was then used to calculate the percentage of the severity of the disease per leaf with the formula severity (%) = (diseased leaf area/ total leaf area)(100) [26].

The software 2LOG [83] was used to design a logarithmic–diagrammatic scale using the principle of Horsfall–Barrat [84,85]. The data on the percentage of the severity of the disease obtained previously were analyzed in the 2LOG computer program to obtain the different estimated classes of the scale, with their confidence limits. The number of classes was determined with respect to the evaluator's measurement ability. In other cases, six classes were recommended, and finally, a representative photograph was associated with each class [26] (Figure 4).

#### 4.6.2. Validation of the Logarithmic—Diagrammatic Scale 

The logarithmic–diagrammatic scale of the pathosystem *C. cassiicola*—*S. lycopersicum* was validated using two evaluations by six participants who estimated the severity of the same 42 leaves and compared them with the classes of the previously elaborated scale. We then compared their estimates with the printed generated scale and the calculated information. Simple linear regressions were used to correlate the values of severity obtained with ImageJ with those estimated by the evaluators. Thus, the precision *r*^2^ and accuracy *b*_1_ of each evaluator was obtained, and with it, the reproducibility of the scale. The *r*^2^ and *b*_1_ values of 1.0 indicated the maximum. SAS ver. 9.4. for Windows was used for all analyses [26]. 

### 4.7. Evaluation of Croton chichenensis Extract on Tomato Leaf Spot

#### 4.7.1. Tomato Crops and Infection with *Corynespora cassiicola*

The *S. lycopersicum* (DR8558) seeds were grown in 200-well polystyrene trays containing Cosmopeat substrate (Cosmocel, Fredericton, NB, Canada) mixed with agrolite (1:1 *v*/*v*). Fifteen days after planting, the seedlings were transplanted into nine-inch pots containing Luvisol rhodic-type soil and irrigated and fertilized as recommended for tomato. To prevent red spider mite (*Tetranychus urticae*) and whitefly (*Bemisia tabaci*) infestation, we used the botanical product BioDi^®^e S.A. (argemonine 3.5% + berberine 2.2% + ricinine 2.8% + α-ter-thienil 3.50%) (Ultraquimia, Benito Juárez, Ciudad de México, México). 

The aqueous extract of *C. chichenensis* roots at 12% (*w*/*v*) was sprayed on the tomato foliage 30 days after transplanting. After 24 h, the plants were inoculated by being sprayed with a conidial suspension of *C. cassiicola* (1 × 10^6^ conidia/mL). 

When the first disease symptoms were observed, 10 leaves per plant were selected from each treatment. Each leaf was compared with the classes of the diagrammatic logarithmic scale previously elaborated and validated. These records were continued at 7-day intervals for three more weeks with the same selected leaves.

#### 4.7.2. Epidemiological Parameters

The disease progress curves were constructed using the severity percentages [16,26] to estimate the AUDPC using the trapezoidal integration method, the apparent infection rate with the model description of Weibull and by its inverse parameter of *b* (1/*b*), and the percentage of disease severity at the end of the evaluations with the scale (*Y*_final_).

### 4.8. Phytotoxicity Bioassay on Solanum lycopersicum Leaf

The toxicity of the aqueous extract of the roots of *C. chichenensis* at doses of 3, 6, and 12% (*w*/*v*) was evaluated on the leaves of *S. lycopersicum*. Ten leaves of 30-day-old *S. lycopersicum* plants were randomly collected and cut into 2 cm^2^ pieces surface, sterilized as described above, and placed in Petri dishes containing agar–agar (BD Becton Dickinson, 23 g L^−1^). The aqueous extract of the *C. chichenensis* roots (50 µL) was deposited on the cut edge of each leaf piece. Sterile distilled water (50 µL) was used instead for the controls [15]. All of the dose treatments were in triplicate and kept at 28 ± 2 °C, 12 h light/12 h dark. Every 24 h for 15 days, samples were examined for any necrosis or darkened areas at the treatment site.

### 4.9. In Vitro Compatibility Bioassay with Beneficial Organisms

*Bacillus subtilis* CBCK47 and *Trichoderma asperellum* Ta13-17 were provided by the Microbiology and Phytopathology Laboratories of the Tecnológico Nacional de México/ Campus Conkal [62,63]. 

The bacteriological agar medium (BD Becton Dickinson, Estado de México, México, 23 g/L), sterile at 50 °C, was mixed with the aqueous extract of the roots of *C. chichenensis* (24%, *w*/*v*) for a final concentration of 12% (*w*/*v*). The agar medium alone was used as the control. Four plates were streaked with *B. subtilis* CBCK47 and incubated at 28 ± 2 °C, with 12 h light/12 h dark. After 48 h, the bacterial colonies were counted [86]. 

For the fungus *T. asperellum* Ta13-17, the aqueous extract from *C. chichenensis* roots was prepared in PDA in plates, as described above, at a final concentration of 12% (*w*/*v*). PDA alone was used as the negative control. The culture medium was inoculated with a mycelial disk (5 mm in diameter) from a 5-day-old colony of *T. asperellum* Ta13-17. The diameter of the colony was then measured every 24 h, as described earlier. The compatibility of the extract with the fungus was estimated using the Abbott formula described above.

## 5. Conclusions

This research is the first contribution aimed at isolating and identifying the phytopathogens associated with tomato leaf spots in the Yucatan Peninsula. Our knowledge of the inhibitory activity of extracts from native species of Yucatan was enriched, in particular, the antifungal spectrum of action of *C chichenensis*. Moreover, we developed a logarithmic–diagrammatic scale for the pathosystem *C. cassiicola–S. lycopersicum* to assess the severity of tomato leaf spots. The aqueous extract of *C. chichenensis* was shown to be a viable alternative to fungicides to reduce the severity of the tomato leaf spots induced by *C. cassiicola* in the greenhouse. The plant extract had no phytotoxicity and was compatible with microbial biocontrol agents, opening an avenue to reduce the use of synthetic fungicides and ensure sustainable control of plant diseases.

## Figures and Tables

**Table 1:** Morphological identification of four phytopathogenic fungi from *Solanum lycopersicum*.

Strain ID	Length (µm)	Width (µm)	Species
ITC24	17–36	7–12	*Alternaria alternata*
ITC23	10–28	1–17	*Corynespora cassiicola*
ITC22	18–32	8–16	*Curvularia lunata*
ITC32	20–83	3–6	*Fusarium equiseti*

**Table 2:** Phytopathogenic fungal strains isolated from *Solanum lycopersicum* with leaf spots.

Strain ID	Source	Molecular Identification		
		Species	Similarity (%) ^a^	GenBank Accession
ITC24	Leaves	*Alternaria alternata*	100	ON815355
ITC23	Leaves	*Corynespora cassiicola*	100	ON815356
ITC22	Leaves	*Curvularia lunata*	100	ON804206
ITC32	Fruit base	*Fusarium equiseti*	99	ON815354

a = Percentage identity with GenBank Blast sequences.

**Table 3:** Inhibition (%) of mycelial growth of four pathogens of *Solanum lycopersicum* by aqueous extracts from native species of the Yucatan Peninsula at 3% (*w*/*v*) in dilution agar assay.

Plant Species	Plant Part	*Alternaria* *alternata* (ITC24)	*Corynespora cassiicola* (ITC23)	*Curvularia lunata* (ITC22)	*Fusarium equiseti* (ITC32)
*Acalypha gaumeri*	root	0 d	7.1 h	1.2 f	0 e
*Bonellia flammea*	stem bark	0 d	27.3 d	93.7 b	8.1 c
*Croton chichenensis*	root	0 d	100 a	99.2 a	100 a
*Calea jamaicensis*	whole plant	0 d	5.7 h	0 g	0 e
*Licaria* sp.	leaves	0 d	2.4 i	22.1 cd	2.3 d
	root bark	0 d	6.5 h	4.9 e	0 e
	stem bark	0 d	15.5 ef	4.8 e	0 e
*Mosannona depressa*	root bark	2.4 c	12.4 g	24.7 cd	7.9 c
	stem bark	35.9 a	48.9 b	19.5 d	44.6 b
*Parathesis cubana*	stem bark	0 d	37.9 c	7.8 e	2.2 d
*Piper neesianum*	leaves	10.2 b	13.8 fg	28.7 c	0 e
Control (water)		0 d	0 j	0 g	0 e

Note: Values with different letters within a column differed significantly in a Tukey test (*p* ≤ 0.05).

**Table 4:** Inhibition (%) of sporulation of fungal pathogens of *Solanum lycopersicum* by aqueous extracts from native species of the Yucatan Peninsula at 3% (*w*/*v*).

Species	Plant Part	*Alternaria* *alternata* (ITC24)	*Corynespora cassiicola* (ITC23)	*Curvularia lunata* (ITC22)	*Fusarium equiseti* (ITC32)
*Acalypha gaumeri*	root	100 a	0 e	100 a	89.9 c
*Bonellia flammea*	stem bark	100 a	83.4 b	100 a	93.3 b
*Croton chichenensis*	root	100 a	100 a	100 a	100 a
*Calea jamaicensis*	whole plants	100 a	10.5 d	0 d	0 f
*Licaria* sp.	leaves	100 a	99.5 a	0 d	49.5 e
	root bark	89.1 b	0 e	90.1 c	0 f
	stem bark	100 a	20.7 c	96.8 b	100 a
*Mosannona depressa*	root bark	27.7 c	21.7 c	100 a	69.9 d
	stem bark	100 a	0 e	100 a	0 f
*Parathesis cubana*	stem bark	0 d	0 e	0 d	0 f
*Piper neesianum*	leaves	27.7 c	21.7 c	100 a	69.9 d
Control (water)		0 d	0 e	0 d	0 f

Note: Values with different letters within a column differed significantly in a Tukey test (*p* ≤ 0.05).

**Table 5:** Inhibition (%) of conidial germination of phytopathogenic fungi with 3% (*w*/*v*) aqueous extracts of plant species.

Species	Plant Part	*Alternaria* *alternata* (ITC24)	*Corynespora* *cassiicola* (ITC23)	*Curvularia lunata* (ITC22)	*Fusarium equiseti* (ITC32)
*Acalypha gaumeri*	root	0.0 b	0.0 f	100 a	100 a
*Bonellia flammea*	stem bark	0.0 b	20.3 d	98.9 a	100 a
*Croton chichenensis*	root	80.9 a	100 a	100 a	70.5 b
*Calea jamaicensis*	whole plants	0.0 b	11.3 e	0.0 c	0.0 d
*Licaria* sp.	leaves	0.0 b	100 a	0.0 c	0.0 d
	root bark	0.0 b	0.0 f	85.2 b	0.0 d
	stem bark	0.0 b	19.9 d	100 a	100 a
*Mosannona depressa*	root bark	0.0 b	80.1 c	100 a	61.3 c
	stem bark	0.0 b	83.7 b	100 a	0.0 d
*Parathesis cubana*	stem bark	0.0 b	0.0 f	0.0 c	0.0 d
*Piper neesianum*	leaves	0.0 b	80.1 c	100 a	61.3 c
Control (water)		0.0 b	0 f	0.0 c	0.0 d

Note: Values with different letters within a column differed significantly in a Tukey test (*p* ≤ 0.05).

**Table 6:** Effect of aqueous extract from *Croton chichenensis* roots (12%, *w*/*v*) on severity of tomato leaf spot 21 days after inoculation with *Corynespora cassiicola* ITC23.

Treatment	AUDPC (%/Day)	*Y*_final_ (%)	Weibull Tasa 1/*b* (%/Day)	*r*^2^ Coefficient of Determination
Extract	64.56 b	10.43 b	0.01901 b	0.98
Control-water	1057.01 a	67.59 a	0.03012 a	0.96

Note: Values with different letters within a column differed significantly in a Tukey test (*p* ≤ 0.05).

**Table 7:** Plants from the Yucatan Peninsula evaluated against pathogenic fungi of tomato.

Species	Family	Site	Voucher	Part
*Acalypha gaumeri* Pax & K. Hoffm	Euphorbiaceae	Yaxcabá	PS-2584	root
*Bonellia flammea* (Millsp. ex Mez) B. Ståhl & Källersjö	Primulaceae	Yaxcabá	PS-2782	stem bark
*Croton chichenensis* Lundell	Euphorbiaceae	Baca	PS-2571	root
*Calea jamaicensis* (L.) L.	Asteraceae	Jahuactal	GC-8084	whole plant
*Licaria* sp.	Lauraceae	Jahuactal	GC-8037	leaves
				root bark
				stem bark
*Mosannona depressa* (Ball.) Chatrou	Annonaceae	Jahuactal	GC-8085	root bark
				stem bark
*Parathesis cubana* (A. DC.) Molinet & M. Gómez	Primulaceae	Jahuactal	JLT-1133	stem bark
*Piper neesianum* C .DC.	Piperaceae	Jahuactal	GC-8080	leaves

## Data Availability

Not aplicable.
